# Supplementary material for: Electrophysiological Activity of Primary Cortical Neuron-Glia Mixed Cultures
Source: Cells. 2023 Mar 6;12(5):821. doi: 10.3390/cells12050821 (PMC10001406; doi:10.3390/cells12050821)
Supplement: Supplementary file 1 [file cells-12-00821-s001.zip › cells-2206398-supplementary.pdf]

cells MDPI

Supplementary Material

Electrophysiological Activity of Primary Cortical Neuron-Glia Mixed Cultures

Noah Goshi [1], Hyehyun Kim [1], Gregory Girardi [1], Alexander Gardner [2] and Erkin Seker [2,*]

[1] Department of Biomedical Engineering, University of California – Davis, Davis CA 95616, USA
[2] Department of Electrical and Computer Engineering, University of California – Davis, Davis, CA 95616, USA
* Correspondence: eseker@ucdavis.edu

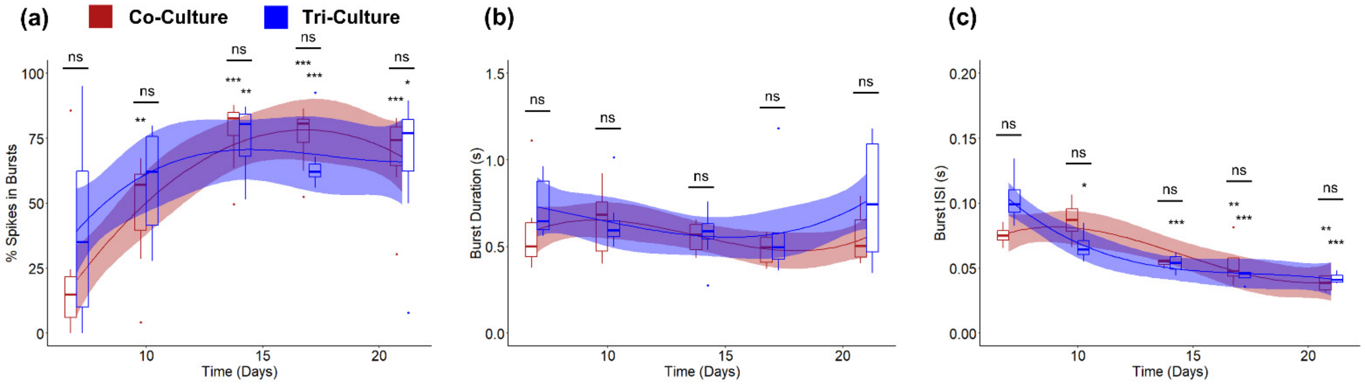

Figure S1. Comparisons of the (**a**) percent spikes in bursts, (**b**) burst duration, and (**c**) interspike interval within bursts between co-cultures (red) and tri-cultures (blue). The solid lines show the fitted linear mixed effects model (treating individual cultures as a random effect) with a b-spline basis. The shaded regions are the 95% confidence interval. An asterisk above an individual box indicates a significant difference of the estimated marginal means of the fitted curves between that timepoint and DIV 7 of the same culture type, while the bars indicate the significance between the co- and tri-culture at that timepoint ($n = 8$, from three independent dissections). * $p < 0.05$, ** $p < 0.01$,

*** $p < 0.001$, ns indicate no significant difference.

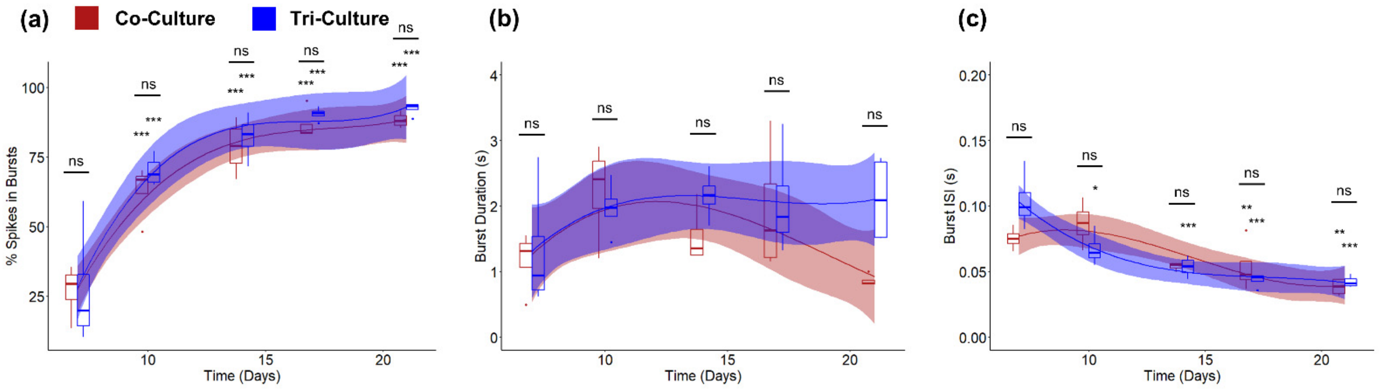

Figure S2. Comparisons of the (**a**) percentage of spikes in bursts, (**b**) burst duration, and (**c**) interspike interval within bursts between co-cultures (red) and tri-cultures (blue) cultured in a two-chambered microfluidic device. The solid lines show the fitted linear mixed effects model (treating individual cultures as a random effect) with a b-spline basis. The shaded regions are the 95% confidence interval. An asterisk above an individual box indicates a significant difference of the estimated marginal means of the fitted curves between that timepoint and DIV 7 of the same culture type, while the bars indicate the significance between the co- and tri-culture at that timepoint ($n = 5$, from two independent dissections). * $p < 0.05$, ** $p < 0.01$, *** $p < 0.001$, ns indicate no significant difference.

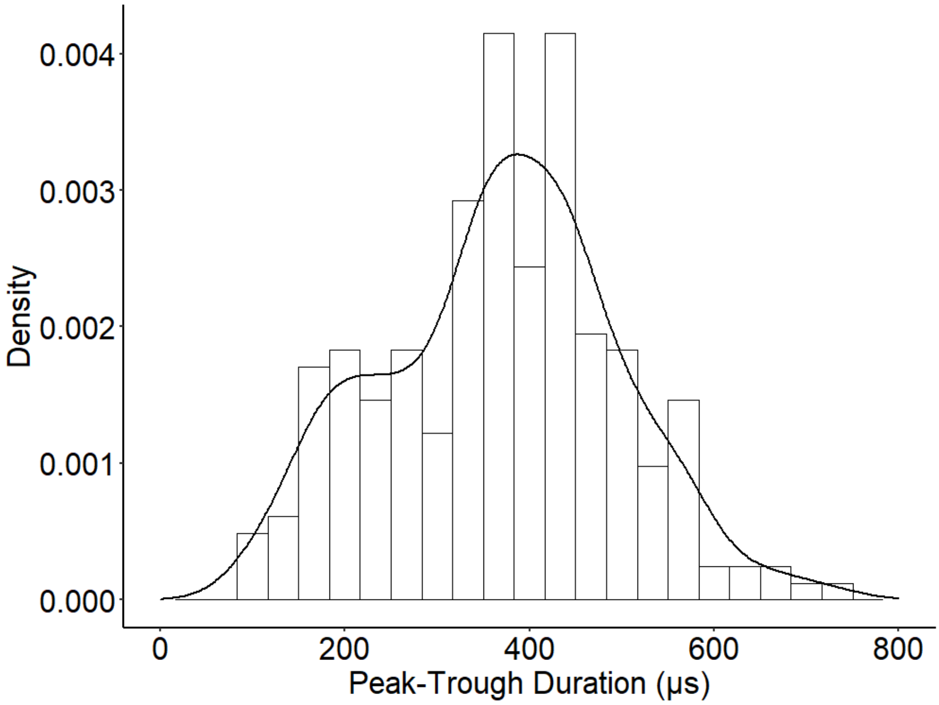

Figure S3. Density histogram of the peak-trough durations of the averaged spike waveforms from 249 units recorded from both co- and tri-cultures. We observe one peak at ~220 μs and the second at ~380 μs.

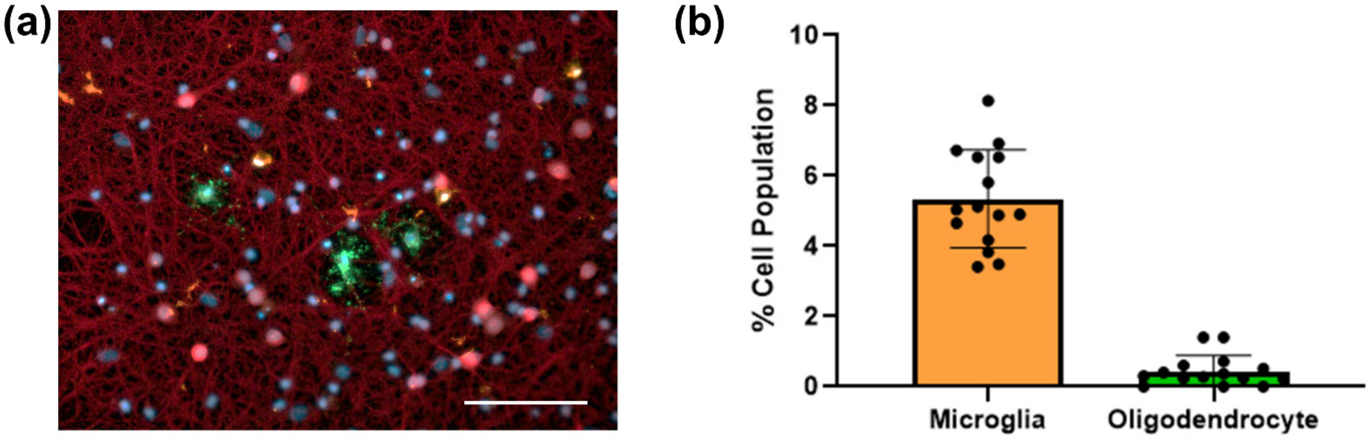

Figure S4. (**a**) Representative fluorescence image of the tri-culture at DIV21 showing the presence of microglia and mature oligodendrocytes. The cultures were stained for neurons - anti-βIII-tubulin (red), mature oligodendrocytes - anti-MBP (green), microglia - anti-Iba1 (orange), and the general nuclear stain DAPI (blue). (Scale bar = 100 µm). (**b**) Percentage of the total cell population of microglia and mature oligodendrocytes. (Mean ± SD, *n* = 3 from one dissection). The individual points indicate the values of the technical replicates.

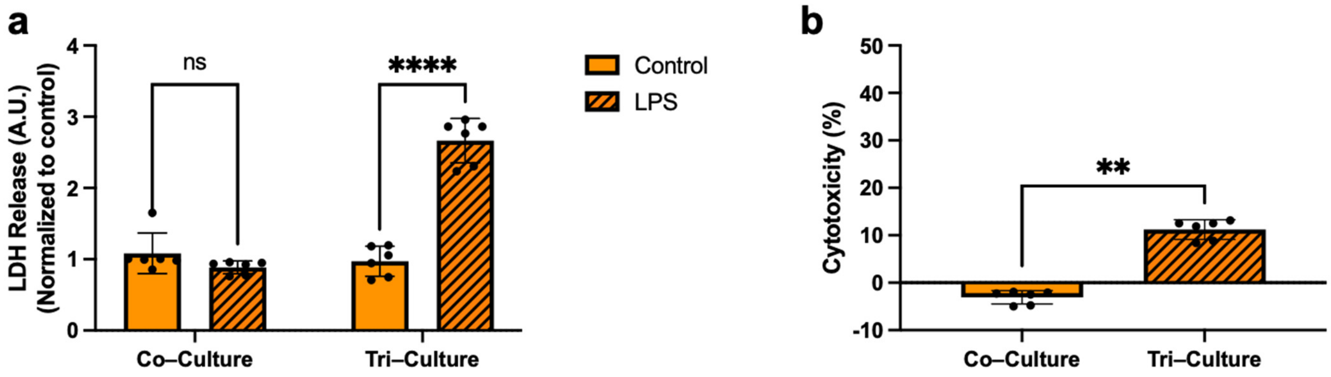

Figure S5. Comparing the change in cytotoxicity between the tri- and co-culture following a 72 h exposure to 5 µg/mL of LPS exposure at DIV 21. Lactate dehydrogenase (LDH) release-based cell viability assessment was conducted using CyQUANT LDH Cytotoxicity Assay Kit (ThermoFisher, Waltham, MA, USA), according to manufacturer's instructions. (**a**) Change in LDH release normalized to controls (vehicle) for each culture type. (**b**) Change in percent cytotoxicity scaled between control (vehicle) and 100% cytotoxicity (lysis buffer treatment). A two-way ANOVA was used to compare the influence of culture type and LPS treatment on cell viability. A Student's t-test was used to compare the differences in percent cytotoxicity between the tri- and co-culture at DIV 21. (Mean ± SD, n = 3 from one dissection). The individual points indicate the values of the technical replicates. ** $p < 0.01$, **** $p < 0.0001$, ns indicate no significant difference.

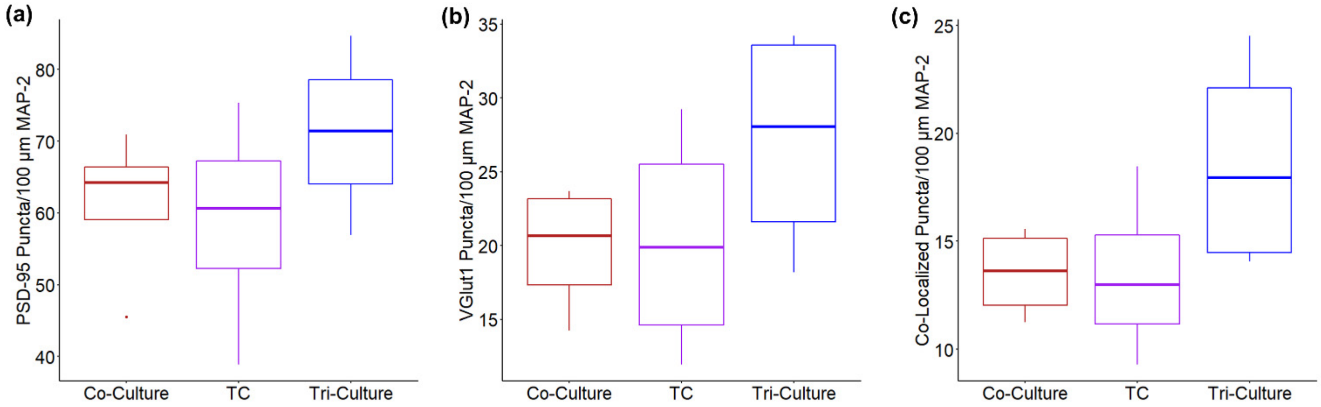

Figure S6. Comparison of the density of (**a**) PSD-95 puncta, (**b**) VGlut1 puncta, and (**c**) co-localized puncta between co-cultures, cultures maintained with co-culture media with the addition of TGF-β (2 ng/mL) and cholesterol (1.5 µg/mL) (TC), and tri-cultures at DIV 21. In all three cases a one-way ANOVA did not find a significant difference between the three culture types. However, qualitatively, the median values of the TC culture are closer to the co-culture than the tri-culture in all three conditions. (n = 4, from two independent dissections).
